# Intranasal Neuropeptide Y as a Potential Therapeutic for Depressive Behavior in the Rodent Single Prolonged Stress Model in Females

**DOI:** 10.3389/fnbeh.2021.705579

**Published:** 2021-09-08

**Authors:** Roxanna J. Nahvi, Arax Tanelian, Chiso Nwokafor, Callie M. Hollander, Lauren Peacock, Esther L. Sabban

**Affiliations:** Department of Biochemistry and Molecular Biology, New York Medical College, Valhalla, NY, United States

**Keywords:** intranasal, neuropeptide Y, females, depression, anxiety, social interaction, posttraumatic stress disorder

## Abstract

The susceptibility to stress-elicited disorders is markedly influenced by sex. Women are twice as likely as men to develop posttraumatic stress disorder (PTSD), depression, anxiety disorders, and social impairments following exposure to traumatic stress. However, most of the studies in animal models examining putative therapeutics for stress-triggered impairments, including single prolonged stress (SPS), were performed predominantly with males. Previous studies in males demonstrated that intranasal neuropeptide Y (NPY) can provide therapeutic relief of many SPS-triggered behaviors, but is ineffective in females at the same dose. Thus, females may need a higher dose of exogenous NPY to attain a therapeutically significant concentration since the overwhelming majority of studies found that NPY levels in females in many brain regions are lower than in male rodents. Here, we examined SPS as an appropriate model to elicit many PTSD-associated symptoms in females and whether intranasal NPY at higher doses than with males is able to alter the development of SPS-triggered behavioral impairments. Sprague-Dawley female rats were exposed to SPS only, or in a separate cohort after SPS stressors were immediately infused intranasally with one of several doses of NPY, starting with 600 μg/rat—four times the dose effective in males. In the third cohort of animals, females were infused intranasally with either 600 μg NPY, omarigliptin [a dipeptidyl peptidase IV (DPP4) inhibitor], or both right after the SPS stressors. After 19 days they were tested on several behavioral tests. SPS elicited significant depressive/despair like behavior on the forced swim test (FST), anxiety behavior on the elevated plus maze (EPM), as well as impaired social interaction. On the FST, there was a dose-response effect of intranasal NPY, with 1,200 μg, but not 600 μg, preventing the development of the SPS-elicited depressive-like behavior. The omarigliptin and 600 μg NPY combined treatment, but neither alone, was also sufficient at preventing depressive-like behavior on the FST. The results demonstrate that: (1) SPS elicits several behavioral manifestations of PTSD in females; (2) early intervention with a high dose of intranasal NPY has therapeutic potential also for females; and (3) NPY cleavage by DPP4 may play a role in the higher dose requirement for females.

## Introduction

Sex is an important factor in the development of stress-triggered disorders. Men are more susceptible to developing substance abuse disorders after experiencing traumatic stress (Kessler et al., 2005). Women, in contrast, are at twice the risk of developing anxiety disorders, depression, and posttraumatic stress disorder (PTSD; Nolen-Hoeksema, 1987; Kessler et al., 1994, 1995; Breslau et al., 1997; Foa and Street, 2001; Breslau, 2009). PTSD is a debilitating and chronic disorder characterized by hyperarousal, avoidance of associated stimuli, re-experiencing of the traumatic event, and changes in cognition or mood (APA, 2013). Notably, major depressive disorder is often comorbid with PTSD (Kessler et al., 2005).

The majority of animal models for PTSD, including the Single Prolonged Stress (SPS) paradigm, have been studied predominantly with males. The SPS model elicits many stress-associated maladaptive behaviors relevant to PTSD, such as anxiety, depression, hyperarousal, impaired social interaction, and impaired fear memory extinction, as well as molecular changes including dysregulation of the HPA axis and activation of the noradrenergic system (Liberzon et al., 1997; Khan and Liberzon, 2004; Kohda et al., 2007; Knox et al., 2012; Eagle et al., 2013; Keller et al., 2015b; Liu et al., 2016; Souza et al., 2017; Lisieski et al., 2018; Nwokafor et al., 2019b; Serova et al., 2019; Mancini et al., 2020). Particularly in the locus coeruleus, the major source for norepinephrine in the brain, SPS induced gene expression of key stress-related enzymes and receptors (Serova et al., 2013b, 2019; Sabban et al., 2015). A few, but not all, recent studies have demonstrated the appropriateness of the SPS model for females in many behavioral and molecular measures, such as the indication of depressive/despair like behavior, anxiety as well as elevation of tyrosine hydroxylase and corticotrophin releasing hormone receptor subtype 1 gene expression in the locus coeruleus (Fan et al., 2013; Keller et al., 2015a; Pooley et al., 2018a,b; Nahvi et al., 2019).

Neuropeptide Y (NPY) is a promising therapeutic for stress-triggered disorders (Sayed et al., 2018; Mathé et al., 2020). Its intranasal application prevented the development and provided relief in many stress-related disorders in males (Serova et al., 2013a,b, 2014; Sabban et al., 2015, 2016; Sabban and Serova, 2018). NPY is found widely throughout the brain, with high expression in the hypothalamus, locus coeruleus, and periaqueductal gray (Kask et al., 2002). There are five known NPY receptors (Y1R, Y2R, Y4R, Y5R, and Y6R), all of which are coupled to G_i_. The actions of NPY receptors include regulating energy homeostasis, bone metabolism, cardiovascular function, epilepsy, and stress. In particular, Y1R activation and Y2R inactivation serve to attenuate depression and anxiety, and there is evidence to suggest Y5R also regulates anxiety behavior (Kask et al., 2002; Benarroch, 2009; Brothers and Wahlestedt, 2010; Reichmann and Holzer, 2016). Furthermore, a Y1R agonist demonstrated superior effects in preventing the development of stress-triggered depressive-like behavior as compared to NPY (Nwokafor et al., 2019a).

There are striking sex differences in NPY expression in unstressed and stressed conditions within many stress-related brain regions and in plasma, with lower expression in females compared to males (reviewed in Nahvi and Sabban, 2020). Indeed, intranasal NPY at concentrations effective in males did not prevent the development of many stress-triggered behaviors in females (Nahvi et al., 2019). It is conceivable that females may need more exogenous NPY for the therapeutic effects considering their lower endogenous levels. Furthermore, NPY is rapidly cleaved by various peptidases and has a half-life of 20 min in plasma, which may be longer in brain tissue (Pernow et al., 1987; Ste Marie et al., 2005; Wagner et al., 2015).

Dipeptidyl peptidase IV (DPP4) is a membrane-bound and shedded soluble peptidase that cleaves NPY, among other peptides. It is found to a large extent in plasma, meninges, and in circumventricular organs (CVOs) such as the median eminence. Its location within CVOs suggests that it plays an important role in NPY regulation at the central nervous system (CNS)-periphery interface. DPP4 is also found on the membranes of microglia and astrocytes to a lesser extent (Wagner et al., 2015). Inhibition of DPP4 results in increased levels of a number of peptides including NPY, glucagon-like peptide-1 (GLP-1), and pituitary adenylate cyclase-activating polypeptide (PACAP; Frerker et al., 2009; Mulvihill and Drucker, 2014; Canneva et al., 2015). DPP4 cleaves the first two N-terminal amino acids of NPY_1–36_, resulting in the NPY_3–36_ which is unable to activate Y1R. This cleavage results in a change of NPY receptor selectivity from a pan-receptor agonist to a Y2R and Y5R selective agonist (Mulvihill and Drucker, 2014; Wagner et al., 2015). The subcellular location of DPP4 is favorable for pharmacologic inhibition as it resides on the membrane surface or is shedded in a soluble form. Gliptins have been used to pharmacologically inhibit DPP4 and are clinically used for diabetes treatment through increasing GLP-1 levels. Most gliptins are given orally and do not cross the blood-brain barrier. However a recent study demonstrated the intranasal application of omarigliptin enhanced brain/plasma ratio of GLP-1, compared to omarigliptin administered orally (Ayoub et al., 2018).

Here we aimed to evaluate in females: (1) effects of SPS on stress-related behavior; (2) a dose response effect and therapeutic dose of intranasal NPY for SPS-elicited behaviors; and (3) the role of NPY cleavage in the high dose requirement of intranasal NPY for treatment of stress-related disorders using the DPP4 inhibitor omarigliptin. The results demonstrate that intranasal NPY at a high dose can provide therapeutic benefit for stress-related behavior, especially depressive/despair behavior, possibly through activation of Y1R.

## Materials and Methods

### Animals

Sprague-Dawley (SD) female rats, about 7 weeks of age, were purchased from Charles River (Wilmington, MA, USA). Animals were maintained on a 12-h light/dark cycle at 22°C with food and water *ad libitum*. They were housed four per cage for at least a week prior to the experiment. All experiments were performed in accordance with the PHS policy and NIH Guide for the Care and Use of Laboratory Animals. All animal studies were approved by the New York Medical College’s Institutional Animal Care and Use Committee (IACUC). They were allowed 2 weeks to accommodate and then randomly assigned to the experimental or control groups.

### Single Prolonged Stress (SPS)

**Figure 1:**
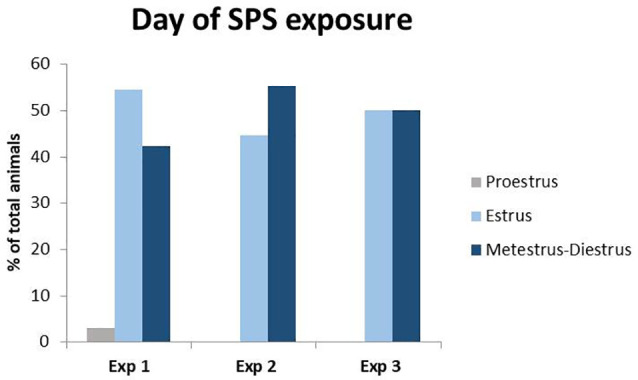
Percent of total animals in each phase of the estrous cycle during Single Prolonged Stress (SPS). Vaginal smears were taken right before the time of SPS in experiments 1–3. Only one animal was in proestrus at the time of SPS in experiment 1 and was excluded from the behavioral analysis to control for fluctuations in estrogen during the proestrus phase.

SPS was performed as previously described (Nahvi et al., 2019) between 8am and 12:30 pm in order to avoid the proestrus phase of the estrous cycle (Figure 1). Briefly, animals were restrained to a custom-made metal board for 2 h and immediately afterward were subjected to a 20 min forced swim in a plexiglass cylinder (50 cm height, 24 cm diameter; Stoelting, Wood Dale, IL, USA) filled two-thirds with 24°C fresh water. Following a 15 min recuperation period, animals were then exposed to diethyl ether in a bell jar until loss of consciousness. Afterwards, animals were housed two per cage and left undisturbed for at least 7 days.

### Administration of Intranasal NPY or DPP4 Inhibitor

Rats while under the influence of ether (the last SPS stressor) were administered a single intranasal infusion of NPY (ABclonal Science Inc., Woburn, MA, USA) freshly dissolved in distilled water and/or 1,200 μg omarigliptin (Selleck Chemicals, Houston, TX, USA) dissolved in 2.5% (*w/v*) sodium dodecyl sulfate. Intranasal omarigliptin or its vehicle was given 20 min prior to administration of intranasal NPY or vehicle in order to physiologically inhibit DPP4 activity. The concentration of omarigliptin administered is reported to inhibit DPP4 by an intranasal route (Ayoub et al., 2018).

A pipetteman with a disposable plastic tip was used to infuse 10 μl of NPY or 50 μl of omarigliptin into each naris. The head was kept tilted back approximately 45° for 15 s to ensure uptake of all of the solution.

### Experiment 1

**Figure 2:**
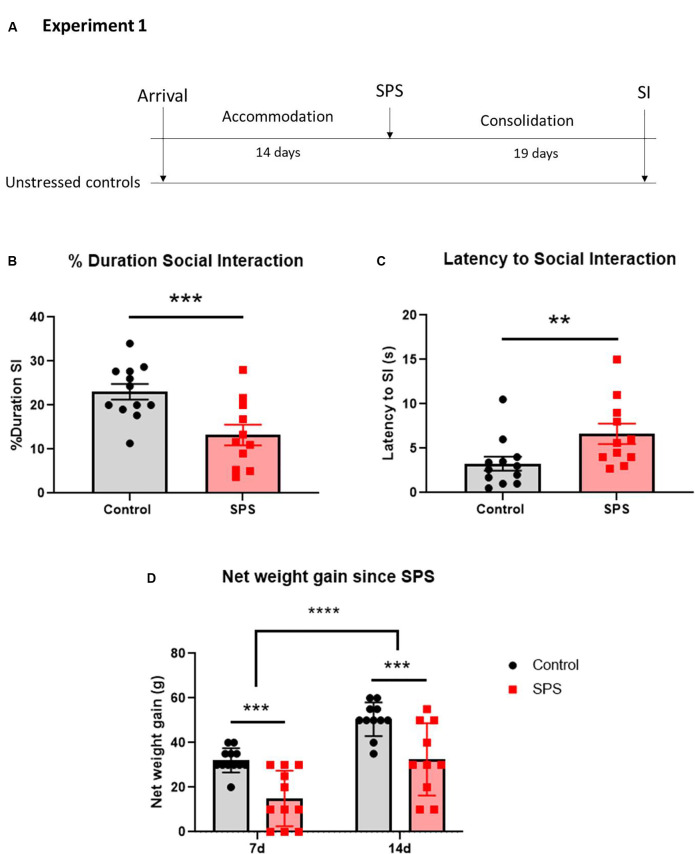
SPS impairs social interaction and weight gain in females. Animals were exposed to SPS stressors and tested for social interaction 19 days afterward, along with unstressed controls **(A)**. The animals were measured for **(B)** percent duration engaged in social interaction and **(C)** latency to the first social interaction encounter. The animals were weighed at 7 and 14 days after SPS and their net weight gain was calculated **(D)**. Each point represents values for an individual animal. Means ± SEM are shown. Unpaired *t*-test and two-way repeated measures ANOVA were performed. Šídák’s multiple comparisons test was used to compare the means between groups and time points. **p < 0.01, ***p < 0.005, ****p < 0.0001.

The experiment design is shown in Figure 2A. Twenty-four 7-week-old female Sprague-Dawley rats (150–170 g) underwent a 14-day accommodation period and were randomly assigned to receive the SPS stressors (*n* = 12) or to the unstressed control group (*n* = 12). The SPS-exposed animals were left undisturbed for at least 7 days to consolidate the experience of traumatic stress. Nineteen days from the time of the SPS stressors, they were tested along with the unstressed controls for social interaction with a juvenile female rat.

### Experiment 2

**Figure 3:**
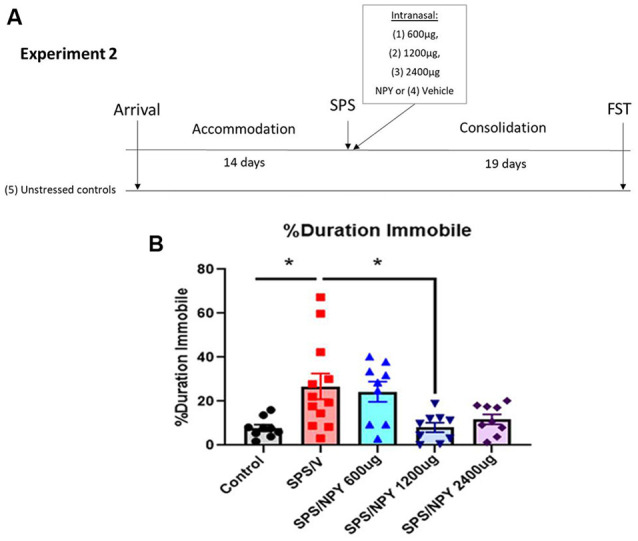
Dose response effect of intranasal NPY on depressive-like behavior. The experimental protocol is shown **(A)**. Animals received one of the increasing doses of intranasal NPY (SPS/NPY_600 μg_; SPS/NPY_1,200 μg_; SPS/NPY_2,400 μg_) or vehicle (SPS/V) immediately after the SPS stressors and were tested on the FST 19 days later, along with unstressed controls **(A)**. The animals were measured for percent duration immobile **(B)**. Each point represents values for an individual animal. Means ± SEM are shown. One-way ANOVA was performed and *post hoc* Tukey was used to determine differences between groups. *p ≤ 0.01.

The experiment design is shown in Figure 3A. Fifty-two 7-week-old female Sprague-Dawley rats (150–170g) were acclimated for 14-days in the animal facility. Forty-two animals underwent SPS and were intranasally infused with either vehicle (SPS/V; *n* = 12), 600 μg NPY (SPS/NPY_600 μg_; *n* = 10), 1,200 μg NPY (SPS/NPY_1,200 μg_; *n* = 10), or 2,400 μg NPY (SPS/NPY_2,400 μg_; *n* = 10) immediately afterward (*n* = 10–12). The animals underwent a consolidation period and were tested on forced swim test (FST) for depressive-like behavior 19 days after the time of SPS together with unstressed controls (*n* = 10).

### Experiment 3

**Figure 4:**
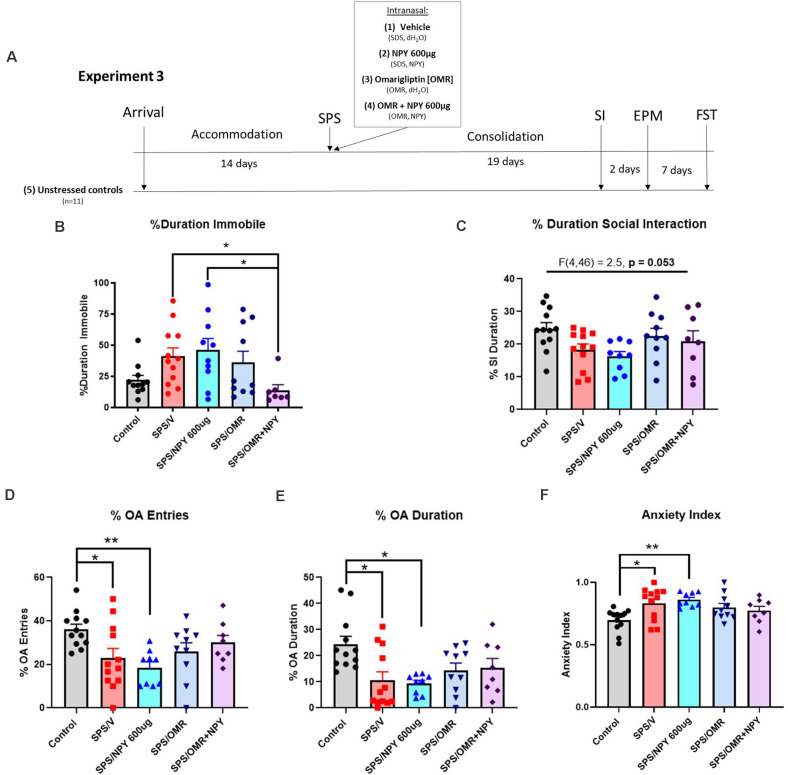
Effects of NPY processing on the high dose requirement for prevention of stress-elicited behavior. The experimental protocol is shown **(A)**. Animals were infused intranasally with either the vehicle for OMR and NPY (SPS/V), NPY_600 μg_ (SPS/NPY_600 μg_), dipeptidyl peptidase IV inhibitor omarigliptin (SPS/OMR), or both (SPS/OMR+NPY) immediately after the SPS stressors. The SPS/NPY_600 μg_ and SPS/OMR groups also received the vehicle for the other drug treatment given as separate infusions (SDS = OMR vehicle; dH_2_O = NPY vehicle). Alongside unstressed controls, the animals were tested for social interaction, anxiety on the EPM, and depressive-like behavior on the FST 19, 21, and 28 days after the SPS stressors, respectively. The animals were measured for **(B)** percent duration immobile on the FST and **(C)** percent duration social interaction. On the EPM, animals were tested for **(D)** percent entries into the open arms (OA); **(E)** percent duration in the OA; **(F)** anxiety index. Each point represents values for an individual animal. Means ± SEM are shown. One-way ANOVA was performed and *post hoc* Tukey was used to determine differences between groups. *p < 0.05, **p < 0.01.

The experiment design is shown in Figure 4A. Fifty-four 7-week-old female Sprague-Dawley rats (150–170 g) were acclimated to the animal facility. Forty-two animals were exposed to the SPS stressors and were given either vehicle (SPS/V; *n* = 12), 600 μg NPY (SPS/NPY_600 μg_; *n* = 10), omarigliptin at 1,200 μg (SPS/OMR; *n* = 10), or both (SPS/OMR+NPY_600 μg_; *n* = 10) intranasally immediately after exposure to ether. In the combined treatment group, NPY was given 20 min after omarigliptin administration while animals were under the influence of ether to allow for physiological inhibition of DPP4 prior to exogenous delivery of NPY. Animals that received only one drug treatment were given the vehicle for the other drug treatment (e.g., SPS/OMR group received omarigliptin and then distilled water 20 min later and SPS/NPY_600 μg_ group received SDS and then NPY 20 min later). Two animals were removed from the SPS/OMR+NPY group as they did not receive the full treatment. After a 19-day consolidation period, animals were tested for social interaction, anxiety on the EPM, and depressive/despair behavior on the FST together with unstressed controls (*n* = 12).

### Social Interaction (SI)

The test animal was placed in the center of the open field arena for 5 min prior to the day of testing and 2 min on the day of testing to habituate, after which a juvenile rat (50–75 g) of the same sex was introduced into the center of the arena. The arena was videotaped. After 5 more min, both animals were removed from the arena and placed in their home cages. The video was analyzed by trained individual blinded to the groups. Time spent in nose-to-nose sniffing, nose-to-anogenital sniffing, following, crawling over and under each other with physical contact, chasing, mounting, and wrestling initiated by the test rat was scored as time spent engaged in social interaction (Varlinskaya and Spear, 2008). The latency time was scored as the duration from the introduction of the juvenile rat into the arena to the first social interaction encounter initiated by the test rat.

### Forced Swim Test (FST)

The FST was performed for 5 min in plexiglass cylinders filled to two-thirds with 24°C fresh water and videotaped. Time spent swimming, defined as the movement of the forelimbs and hind limbs, and the time spent immobile when the animal showed no movement, or only movements needed to keep its head above the water were scored by a trained individual blinded to the experimental groups.

### Elevated Plus Maze (EPM)

The EPM was performed in a room with dim light as previously described (Serova et al., 2013b). Animals were allowed to acclimate to the room for 30 min before the test. Each rat was placed on the central platform facing an open arm and was allowed to explore the maze for 5 min. The behavior was recorded (Biobserve) and a trained individual blinded to the experimental groups measured the duration and entries into the open and closed arms (OA and CA, respectively). We calculated the anxiety index as 1 − [(time spent in OA/total time on the maze)/2 + (number of entries to the OA/total exploration on the maze)/2].

### Vaginal Smears

Vaginal smears were collected and analyzed as described in McLean et al. (2012) with some modifications. In brief, a vaginal swab was taken using autoclaved distilled water and the collected specimen was allowed to dry on a glass slide. The slides were stained with 0.1% crystal violet and observed under a light microscope at 10× and 40× magnification. Views from three distinct sections of the vaginal smear were analyzed to determine the phase of the estrous cycle by a trained investigator blinded to the groups.

Vaginal smears were taken on the day of SPS, as well as the day of the behavioral assessment. Since estrogen has stress-protective effects and peaks during the proestrus phase of the estrous cycle (Jaric et al., 2019; Frye et al., 2000; Marcondes et al., 2001), all experiments were concluded by the early afternoon, the start of time proestrus. Analysis revealed (Figure 1) that only one female was in the proestrus phase of the estrous cycle during either exposure to the SPS stressors or behavioral testing. This animal was removed from the analyses to control for differences in proestrus-related increases in estrogen.

### Statistical Analysis

Data analysis was performed in Prizm 9 (GraphPad) software. Normality tests were done using D’Agoustino and Pearson Omnibus. Data were analyzed by t-test or Mann-Whitney test for comparison of the means between two groups; one-way ANOVA with *post hoc* Tukey test or the Kruskal-Wallis test with Dunn’s multiple comparison test for Gaussian or non-Gaussian distributions, respectively; or two-way repeated measures ANOVA with *post hoc* Šídák’s multiple comparisons test for comparison of the means from different time points. Outliers were removed when they were greater than two standard deviations away from the mean. Values at *p* ≤ 0.05 were considered significant.

## Results

The effect of SPS on social interaction in female rats was examined (Figure 2). The females were exposed to SPS or untreated, and after 19 days were tested for social interaction (Figure 2A). The results revealed that SPS induced social impairment in females. SPS-exposed females spent significantly less time engaging in social interaction than unstressed controls (*t*_(21)_ = 3.39, *p* = 0.0028; Figure 2B). Additionally, the SPS group had a longer latency to first approach the other animal (*U* = 24, *p* = 0.008; Figure 2C).

As another measure of the stress response, animal body weight measurements were taken at the time of the SPS stressors, as well as 7 and 14 days afterward and the net weight gain from the day of the SPS stressors was calculated. Two-way ANOVA with repeated measures revealed significant differences in net weight gain both between the groups (*F*_(1,21)_ = 16.5, *p* = 0.006) and over time (*F*_(1,19)_ = 82.32, *p* < 0.0001), with no significant interaction. SPS-exposed females had a lower net weight gain than unstressed controls 7 (*p* = 0.0012) and 14 (*p* = 0.0018) days after the time of SPS. Furthermore, the net weight gain increased 14 days after SPS as compared to 7 days in both the SPS (*p* < 0.0001) and unstressed control (*p* < 0.0001) groups (Figure 2D). There were no differences in the net weight gain from 7–14 days after SPS compared to the unstressed controls (data not shown), indicating the most severe effects of the stress on body weight occurred in the first week following exposure to the SPS stressors.

In experiment 2 (Figure 3), we evaluated the dose-response effect of intranasal NPY starting at 600 μg/rat to alter the development of depressive-like behavior on the FST 19 days after exposure to the SPS stressors (Figure 3A). One-way ANOVA revealed significant differences in the percent duration immobile on the FST (*F*_(4,43)_= 5.2, *p* = 0.0017). Tukey comparison showed SPS/V animals spent more time immobile than unstressed controls (*p* = 0.01). NPY at 1,200 μg, but not 600 μg, significantly prevented the development of the SPS-elicited increase in immobility (*p* = 0.01; Figure 3B).

In experiment 3, to investigate the involvement of NPY cleavage and indirectly evaluate the importance of Y1R activation, females were infused intranasally with 600 μg NPY, 1,200 μg omarigliptin, or both immediately after the SPS stressors (Figure 4A). On the FST, there were significant differences in the percent duration immobile (*H*_(4)_ = 11.88, *p* = 0.018). Only the combined OMR+NPY_600 μg_ treatment significantly prevented the development of depressive-like behavior on the FST (*p* = 0.03). Furthermore, the group that received the combined therapy spent significantly less time immobile than the SPS/NPY_600 μg_ treated animals (*p* = 0.038). Animals that received omarigliptin alone did not statistically differ from the SPS/V or SPS/NPY_600 μg_ treated groups (Figure 4B).

The same cohort of animals from experiment 3 was also tested for social interaction and on the EPM for anxiety-like behavior 19 and 21 days after exposure to the SPS stressors, respectively (Figure 4A). One-way ANOVA analysis of the percent duration of social interaction was near significant (*F*_(4,46)_ = 2.5, *p* = 0.053). Neither NPY_600 μg_, omarigliptin, nor the combined therapy was sufficient at preventing SPS-elicited social impairment in females (Figure 4C).

SPS elicited anxiety on all EPM measures, and intranasal treatment was not sufficient to prevent its development. There were significant differences in the percent of OA entries among the groups (*F*_(4,46)_ = 3.8, *p* = 0.0095). Compared to unstressed controls, animals in the SPS/V (*p* = 0.0485) and SPS/NPY_600 μg_ (*p* = 0.0076) group had significantly fewer entries into the OA (Figure 4D). Additionally, one-way ANOVA revealed significant differences in the time spent in the OA (*F*_(4,46)_ = 4.4, *p* = 0.0043). Both the SPS/V (*p* = 0.0074) and SPS/NPY_600 μg_ (*p* = 0.0067) treated animals spent significantly less time in the OA than unstressed controls (Figure 4E). There were also significant differences in the overall anxiety index (*F*_(4,46)_ = 4.3, *p* = 0.005). The SPS/V (*p* = 0.016) and SPS/NPY_600 μg_ (*p* = 0.005) treated animals had a higher anxiety index than unstressed controls (Figure 4F). The SPS/OMR and SPS/OMR+NPY_600 μg_ treated group did not differ from SPS/V or unstressed controls in the percent of OA entries and duration or their anxiety index (Figures 4D–F).

## Discussion

The results of this study demonstrate that SPS can be an appropriate model for studying at least some of the features of PTSD in females and that intranasal NPY at a high dose can provide early therapeutic intervention, likely as a Y1R agonist. As in males, in females SPS elicited many PTSD maladaptive behaviors, such as anxiety, depressive/despair behavior, and impaired social interaction in females. Intranasal NPY at a high dose effectively prevented the development of depressive-like behavior on the FST in females. Furthermore, the DPP4 inhibitor enabled a normally ineffective low dose of intranasal NPY to prevent the development of depressive-like behavior on the FST. This suggests that the high dose requirement of intranasal NPY may be due in part to its cleavage to a peptide not recognized by the Y1R receptor.

### Single Prolonged Stress as an Appropriate Stress Model for Some PTSD Associated Symptoms in Females

It is important to have an appropriate preclinical model of PTSD and associated disorders for females. SPS has previously been demonstrated to elicit indications of anxiety, depression, allodynia, and hyperalgesia in females (Pooley et al., 2018a,b; Zhang et al., 2018; Nahvi et al., 2019). Here, we similarly found that exposure to SPS stressors increases anxiety on the EPM. It also led to immobility on the FST, an indication of depressive-like behavior or passive coping. Furthermore, this is the first study to report changes in weight gain and impaired social interaction 2 weeks or more after the time of SPS in females.

There are conflicting reports of whether or not SPS is appropriate for studying fear memory impairments in females. Stressed females exhibited increased acquisition of contextual fear compared to unstressed controls 7 and 14 days after the SPS stressors (Fan et al., 2013; Zer-Aviv and Akirav, 2016). Furthermore, SPS-exposed females were unable to extinguish contextual fear memory, however did not differ from unstressed controls in retention of extinguished cued fear memory (Keller et al., 2015a; Zer-Aviv and Akirav, 2016). In males, SPS impaired fear extinction of both contextual and cued fear conditioning 7 days after the stressors (Knox et al., 2012).

Moreover, males and females are reported to exhibit a diverging response to the acoustic startle response (ASR), a measure of hyperarousal, after exposure to SPS stressors. SPS-exposed males displayed hyperarousal compared to unstressed controls, whereas similarly stressed females and their unstressed counterparts did not differ in their ASR (Pooley et al., 2018a,b).

While we found that SPS elicited anxiety behavior 21 days after exposure to the stressors, there are variable results when measured 30 days afterward. Zhang et al. (2018) found overall increased anxiety 30 days after SPS exposure in females, whereas Mancini and colleagues reported no difference in anxiety behavior between SPS-exposed and unstressed females. Interestingly, stressed males exhibited increased anxiety at this time point compared to unstressed controls (Mancini et al., 2020).

Housing may play a role in stress susceptibility between females and males. In our studies with SPS in both males and females, they were pair housed after SPS. Females are reported to be more susceptible to stress when housed individually, whereas males generally appear to be more stressed in group housing conditions. When placed in overcrowded housing, males had elevated corticosterone levels more than 1 week afterward and exhibited increased anxiety behavior after 24 h (Brown and Grunberg, 1995; Palanza et al., 2001). Conversely, individually housed females demonstrated higher circulating corticosterone levels and anxiety behavior than their grouped housed counterparts (Brown and Grunberg, 1995; Palanza et al., 2001). Females were more sensitive to housing-dependent changes in anxiety behavior, particularly those in non-proestrus phases (Palanza et al., 2001). Additionally, singly housed females exposed to SPS stressors had a shorter latency to approach a novel rat, than pair-housed females (Pooley et al., 2018b). Females may be more dependent on social support in stressful conditions, while males may be more influenced by social hierarchy and dominance. Thus, housing conditions are extremely important to consider in developing stress models for males and females, as the social environment influences biological and behavioral indices of stress in each sex differently.

Another major challenge to representing PTSD- and stress-associated symptoms in female animal models is the variable methods used to control for and analyze the effects of the estrous cycle. In our studies, we focused on avoiding proestrus during the SPS stressors and behavioral testing as it is generally known to have stress-protective effects due to the increase in circulating estrogen and progesterone during this phase (Jaric et al., 2019; Frye et al., 2000; Marcondes et al., 2001). In addition, we did not want to disturb or stress the animals, so vaginal smears were only taken when they were exposed to SPS or after behavioral tests. Other studies examining SPS as an appropriate traumatic stress model for females selected animals only in diestrus (Zer-Aviv and Akirav, 2016), phase-matched animals in each experimental group (Keller et al., 2015a), or had no estrous cycle selection criteria for SPS exposure (Pooley et al., 2018a,b). Additionally, variations in the estrous cycle on the day of behavioral testing can influence results. Unstressed females in proestrus have less anxiety and depressive-like behavior on the EPM and FST compared to the other estrous cycle phases (Contreras et al., 2000; Marcondes et al., 2001). Moreover, females displayed superior retention of extinguished fear in proestrus than those in metestrus (Milad et al., 2009). Therefore, it is crucial that investigators are attentive to the influence of the estrous cycle and hormone fluctuations both during stress exposure and behavioral testing.

It is also important to consider the effects of estrogen and the estrous cycle when investigating the stress response and NPY system in females. Y1R gene expression is up-regulated by estrogen. Hypothalamic Y1R expression increases in the proestrus, or the “high-estrogen”, phase of the estrous cycle (Martini et al., 2011) and the Y1R promotor contains estrogen response elements (Eva et al., 2006). Furthermore, estrogen increases NPY mRNA expression, NPY-immunostaining neurons, and NPY release in the hippocampus (Hilke et al., 2009; Veliskova et al., 2015). Animals in proestrus also have higher NPY mRNA expression levels in the arcuate nucleus than a “low-estrogen” phase of the estrous cycle (Bauer-Dantoin et al., 1992). Therefore, careful attention to the estrous cycle, particularly the proestrus phase, is needed when examining the effects of stress and NPY response in females.

### Effects of Intranasal Neuropeptide Y in Females

The findings from this study can help clarify sex differences in response to intranasal NPY for the treatment of stress-elicited disorders. Previous studies in males demonstrated that intranasal NPY at 150 μg was sufficient to prevent the development of anxiety and depressive-like behavior (Serova et al., 2013b), however, this was unsuccessful in females (Nahvi et al., 2019). Here we found that a high dose of intranasal NPY (8× the therapeutic concentration used in males) was effective at preventing depressive-like behavior on the FST in females.

A preponderance of literature report that females have lower NPY expression in many stress-related brain regions both in unstressed and stressed conditions (reviewed in Nahvi and Sabban, 2020). Studies show females have relatively lower NPY peptide levels in the hypothalamus, hippocampus, striatum, and plasma in unstressed conditions (Rugarn et al., 1999; Ruohonen et al., 2008). Stressed-exposed females have lower NPY peptide levels in the basolateral amygdala, hippocampus, and in plasma than their stressed male counterparts (Zukowska-Grojec, 1995; Miragaia et al., 2018). Here, females needed a higher dose of exogenous NPY to attain a therapeutically significant concentration or the NPY system may not be as relevant for stress- related disorders in females compared to males.

This becomes especially important as females have a hyper-reactive locus coeruleus in response to CRH. Females demonstrate increased surface CRHR1 expression and increased dendritic density receiving input from the limbic system compared to males (Curtis et al., 2006; Bangasser and Valentino, 2014). Greater activation of the locus coeruleus in response to CRH and the limbic system coupled with failure to increase endogenous NPY expression in response to stress may result in a more severe stress response. Consequentially, females may inherently require more NPY to abate CRH-induced stress effects.

There is a link between NPY and depressive-like behavior in both sexes. The Flinders Sensitive Line (FSL) rat is a widely used animal model for depression with the Flinders Resistant Line (FRL) as its control. In both females and males, the FSL animals had less NPY peptide expression in the hippocampus than their FRL controls. Interestingly, both FSL and FRL females had lower NPY expression than their male counterparts (Jimenez-Vasquez et al., 2000). Furthermore, there was a positive correlation between hippocampal NPY mRNA expression and duration swimming on the FST in FSL females (Bjørnebekk et al., 2010). Global knockout of NPY increased depressive-like behavior on the FST in both males and females (Painsipp et al., 2010b). Thus, NPY appears to play a role in regulating depressive-like behavior also in females, which is consistent with our findings.

The need for high doses of intranasal NPY in females may be in part due to the rapid degradation of the peptide. NPY can be cleaved by many proteases, including dipeptidyl peptidase IV (Pernow et al., 1987; Wagner et al., 2015). Application of a DPP4 inhibitor would increase the amount of exogenously administered NPY_1–36_. DPP4-deficient rats exhibit increased NPY levels in the CSF, as well as improved fear extinction (Canneva et al., 2015). Interestingly, DPP4-deficient rats demonstrated lower expression of the glucocorticoid receptor (GR) and FKBP5, a chaperone protein for GR, in the amygdala and hypothalamus as well as less stress-induced peripheral corticosterone levels, but did not differ from wildtypes in the abundance of NPY-ergic neurons in the basolateral amygdala, dentate gyrus, and CA1 region of the hippocampus (Golub et al., 2019).

The location of DPP4 within CVOs, particularly the median eminence, and the meninges has implications for DPP4-targeted intranasal manipulations on stress-related behaviors. The median eminence, a hypothalamic nucleus, releases CRH upon stress which then promotes the HPA axis and stress response (Vernikos-Danellis, 1965), and it contains CRH/Y1R co-localized neurons (Dimitrov et al., 2007). The action of Y1R, which is generally inhibitory (Benarroch, 2009), in the median eminence, may serve to reduce synaptic firing and release of CRH that would otherwise promote the HPA axis. Furthermore, intranasally delivered drugs reach the subarachnoid meningeal space where CSF is housed and is distributed throughout the brain (Dhuria et al., 2010). Thus, DPP4 interactions with NPY may play a critical stress-regulating role in these CNS regions.

In this study, we found that the intranasal omarigliptin and NPY_600 μg_ combined therapy was sufficient to prevent the development of depressive-like behavior on the FST. The group that received the combined therapy has less immobility on FST than the SPS/V and SPS/NPY_600 μg_ groups, whereas the SPS/OMR group did not significantly differ from either. This suggests that the resulting anti-depressive effects are not due to an increase of other DPP4 substrates and that DPP4 inhibition alone is not sufficient to produce anti-depressive effects in stress-exposed females. This could be due to a need for exogenous NPY administration as a result of low endogenous NPY expression. Furthermore, it suggests the sub-therapeutic dose of NPY 600 μg can be effective at preventing depressive-like behavior with an adjunctive DPP4 inhibitor.

It has been reported that the anti-depressant effects of NPY act through Y1R (Brothers and Wahlestedt, 2010; Nwokafor et al., 2019a). The application of a DPP4 inhibitor would cause NPY to maintain Y1R agonism by preventing its cleavage to the Y2R and Y5R selective agonist, NPY_3–36_. Therefore, it appears that the anti-depressive effects of intranasal NPY in females can be attributed to action on Y1R.

**Figure 5:**
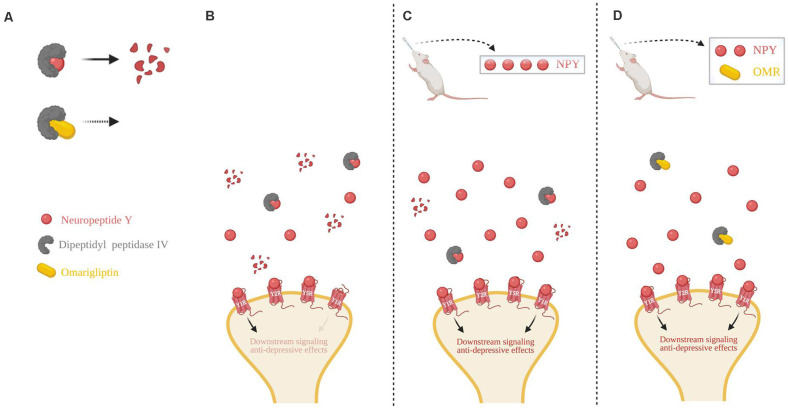
Schematic of intranasal NPY treatment and role of NPY cleavage in females. **(A)** The first 2 N-terminal amino acids of NPY are cleaved by dipeptidyl peptidase IV (DPP4), changing it from a pan-receptor agonist to a Y2R and Y5R selective agonist. Omarigliptin (OMR) blocks the activity of DPP4, preventing NPY’s cleavage. **(B)** Without any exogenous delivery of the drug, DPP4 cleaves NPY resulting in a low concentration of the full-length NPY peptide. **(C)** Delivery of a high dose of intranasal NPY rapidly reaches the central nervous system and results in a higher concentration of the full-length NPY peptide in the synapse, despite some cleavage by DPP4. **(D)** Intranasal administration of OMR and a lower dose of NPY results in a similar concentration of the full-length NPY peptide as compared to delivery of a high dose of intranasal NPY in part due to the inhibition of its cleavage. Higher synaptic availability of the full-length NPY peptide is likely able to have greater activation of Y1R, compared to the NPY cleavage product. Illustration created with BioRender^©^.

We propose a schematic to explain the findings in this study in conjunction with the literature (Figure 5). Cleavage of NPY by DPP4, which results in loss of action at Y1R, can be inhibited by omarigliptin (Figure 5A). In basal conditions with no exogenous delivery, there are low endogenous levels of NPY in the synapse, as well as the presence of proteolytic DPP4 resulting in less Y1R binding and weaker anti-depressive effects (Figure 5B). A high dose of intranasal NPY increases the bioavailability of full-length NPY in the synapse which would lead to an increased probability of ligand-to-receptor binding, including Y1R, and result in stronger anti-depressive actions (Figure 5C). When administered a sub-therapeutic dose of NPY in combination with the DPP4 inhibitor omarigliptin, more full-length NPY is available in the synapse, thus increasing the probability of NPY binding to its receptors, particularly Y1R, leading to therapeutic anti-depressive effects. Therefore, less exogenous NPY is needed to attain therapeutic benefit (Figure 5D).

In this study, in contrast to depressive behavior, sub-therapeutic dose of NPY was not sufficient to prevent the development of SPS-elicited anxiety-like behavior on any of the EPM measures or social impairments. Thus, the combined therapy and omargliptin alone did not significantly prevent the development of stress-elicited anxiety-like behavior or social anxiety in females. This can indicate that NPY may not be an effective therapy for preventing anxiety-like behavior and social anxiety in females or that perhaps a higher dose is required.

In fact, a higher dose of selective serotonin reuptake inhibitors (SSRIs) appears to be required to clinically treat anxiety disorders than depression (Cassano et al., 2002). Moreover, ICV administration of NPY directly into the lateral ventricle was anxiolytic in females (Badia-Elder et al., 2003). Germinal inactivation of NPY in females revealed no difference in anxiety-like behavior on the EPM or the light-dark anxiety tests, however, displayed increased anxiety behavior on the open field test (Karl et al., 2008; Painsipp et al., 2011). Overall, it appears that NPY can regulate anxiety in females, however, it may be dose or site-specific.

Knockout studies reveal further insight on the implications of NPY receptors in the regulation of depressive- and anxiety-like behavior in females. Female Y1R germinal knockout mice exhibited no difference in depressive- or anxiety-like behavior under unstressed conditions, however, this may be dependent on the light/dark cycle (Karl et al., 2006), limbic system-specific inactivation of Y1R (Longo et al., 2014; Bertocchi et al., 2020), or exposure to a stressor (Painsipp et al., 2010b). However, a combined cohort of male and female Y1R knockout mice had increased immobility, or higher depressive-like behavior, on the FST than WT controls (Karlsson et al., 2008). Depressive- and anxiety-like behavior was decreased with germinal Y2R and Y4R inactivation in females (Painsipp et al., 2008a,b, 2010a), however, one study reported Y2R knockout had no effect on depressive-like behavior (Zambello et al., 2011). It is important to note these knockout studies examine the effects of global elimination of different NPY receptors.

Furthermore, human studies suggest that NPY delivered intranasally can demonstrate therapeutic effects for stress-related disorders in females and in a dose-dependent fashion. In one study, intranasal NPY at high doses was shown to be anxiolytic in PTSD patients, 66% of which were female (Sayed et al., 2018). A separate study found that a single infusion of intranasal NPY acutely improved depressive symptoms in patients with major depressive disorder, where more than 50% of the participants were female (Mathé et al., 2020). These promising clinical studies demonstrate the translational stress-protective effects of intranasal NPY in females and that perhaps high concentrations or repeated doses may be required.

## Limitations

There are some limitations that temper the conclusions from this study. It is clear that SPS can be an appropriate model for females, at least for several behaviors, and that NPY can be therapeutic in females at high doses, at least for depressive/despair behavior. However, the suggestions regarding sex differences need to be confirmed since the males and females were not studied in parallel.

For example, these studies assume that uptake of NPY from the nasal cavity through the olfactory and trigeminal nerves to the brain does not differ between males and females. Preliminary data from our lab revealed no difference in the cerebrospinal fluid NPY concentration between males and females 30 min after its intranasal delivery when given the same dose. However, further studies are needed to assess any sex differences in intranasal NPY pharmacokinetics and pharmacodynamics.

Lastly, the proestrus phase was avoided in the tested females to control for the peak in estrogen and other ovarian hormones, which are known to affect the stress response and NPY expression (Frye et al., 2000; Marcondes et al., 2001; Molina-Hernández et al., 2006; Hilke et al., 2009; Veliskova et al., 2015; Jaric et al., 2019). While this controls for fluctuations in ovarian hormones, future studies should use larger cohorts of animals and phase-match for improved translational validity.

## Conclusion

Overall the results demonstrate that SPS elicits social impairment, depressive-like and anxiety behaviors, as well as stress-elicited impairments in weight gain in females. Furthermore, intranasal NPY can be an effective therapeutic for the prevention of behavioral impairment in females at a high dose. The higher dose needed to prevent the development of stress-triggered depressive-like behavior may be due to lower endogenous NPY expression in females, compared to males. There is some effect of NPY cleavage by DPP4 on the high dose requirement, evident by the reduction of the sufficient NPY dose with the application of a DPP4 inhibitor. Furthermore, NPY receptor selectivity may play an important role in its anti-depressive action, with Y1R likely exerting a major effect.

## Data Availability Statement

The raw data supporting the conclusions of this article will be made available by the authors, without undue reservation.

## Ethics Statement

The animal study was reviewed and approved by New York Medical College.

## Author Contributions

RN and ES planned the study and wrote the manuscript. RN, AT, CN, CH, and LP performed the experiments and data analysis. All authors read and approved the manuscript. All authors contributed to the article and approved the submitted version.

## Conflict of Interest

The authors declare that the research was conducted in the absence of any commercial or financial relationships that could be construed as a potential conflict of interest.

## Publisher’s Note

All claims expressed in this article are solely those of the authors and do not necessarily represent those of their affiliated organizations, or those of the publisher, the editors and the reviewers. Any product that may be evaluated in this article, or claim that may be made by its manufacturer, is not guaranteed or endorsed by the publisher.
